# KIF11 promotes vascular smooth muscle cell proliferation by regulating cell cycle progression and accelerates neointimal formation after arterial injury in mice

**DOI:** 10.3389/fphar.2024.1392352

**Published:** 2024-08-06

**Authors:** Gengqiao Wang, Peng Zhao, Chuanzheng Yin, Xichuan Zheng, Yuhang Xie, Xuan Li, Dan Shang, Shuyu Shao, Hao Chen, Li Wei, Zifang Song

**Affiliations:** ^1^ Department of Hepatobiliary Surgery, Union Hospital, Tongji Medical College, Huazhong University of Science and Technology, Wuhan, Hubei, China; ^2^ Department of Vascular Surgery, The Southwest Hospital of AMU, Chongqing, China; ^3^ Department of Vascular Surgery, Union Hospital, Tongji Medical College, Huazhong University of Science and Technology, Wuhan, Hubei, China; ^4^ Department of Gerontology, Union Hospital, Tongji Medical College, Huazhong University of Science and Technology, Wuhan, Hubei, China

**Keywords:** KIF11, VSMCs, vascular injury, cell proliferation, neointima formation

## Abstract

**Background and aims:** One of the primary causes of lumen narrowing is vascular injury induced during medical procedures. Vascular injury disrupts the integrity of the endothelium, triggering platelet deposition, leukocyte recruitment, and the release of inflammatory factors. This, in turn, induces the proliferation of vascular smooth muscle cells (VSMCs), leading to neointima formation. However, the molecular mechanism underlying VSMC proliferation following injury remains unknown. KIF11 is critical in regulating the cell cycle by forming bipolar spindles during mitotic metaphase. This process may contribute to VSMCs proliferation and neointima formation following vascular injury. Yet, the function of KIF11 in VSMCs has not been elucidated. This study aims to investigate the role and mechanisms of KIF11 in regulating VSMCs cycle progression and proliferation.

**Methods:** After conducting biological analysis of the transcriptome sequencing data from the mouse carotid artery injury model and the cell transcriptome data of PDGF-BB-induced VSMCs, we identified a potential target gene, KIF11, which may play a crucial role in vascular injury. Then we established a vascular injury model to investigate how changes in KIF11 expression and activity influence *in vivo* VSMCs proliferation and neointimal formation. In addition, we employed siRNA and specific inhibitors to suppress KIF11 expression and activity in VSMCs cultured *in vitro* to study the mechanisms underlying VSMCs cycle progression and proliferation.

**Results:** The results of immunohistochemistry and immunofluorescence indicate a significant upregulation of KIF11 expression in the injured vascular. The intraperitoneal injection of the KIF11 specific inhibitor, K858, partially inhibits intimal hyperplasia in the vascular injury model. *In vitro* experiments further demonstrate that PDGF-BB upregulates KIF11 expression through the PI3K/AKT pathway, and enhances KIF11 activity. Inhibition of both KIF11 expression and activity partially reverses the pro-cycle progression and pro-proliferation effects of PDGF-BB on VSMCs. Additionally, KIF11 overexpression partially counteracts the proliferation arrest and cell cycle arrest induced by inhibiting the PI3K/AKT pathway in VSMCs.

**Conclusion:** Our study highlights the crucial role of KIF11 in regulating the cycle progression and proliferation of VSMCs after vascular injury. A comprehensive understanding of these mechanisms could pave the way for potential therapeutic interventions in treating vascular stenosis.

## Graphical Abstract

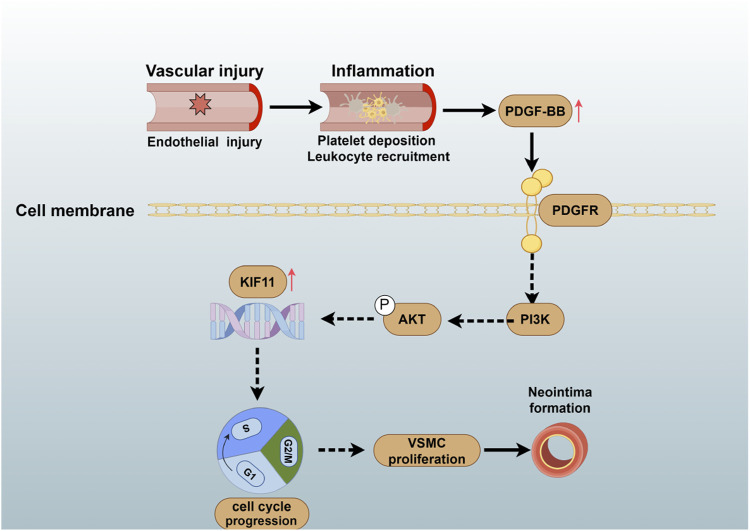

## 1 Introduction

Vascular injury is one of the primary causes of vascular luminal stenosis. Vascular injury stimulates platelet aggregation, promotes the secretion of growth factors and inflammatory factors, induces phenotypic changes in VSMCs, and regulates the cycle, proliferation, and migration of VSMCs (Shah, 2003; Muto et al., 2013; Wu et al., 2017; Frismantiene et al., 2018). This ultimately leads to the neointima formation and lumen narrowing or occlusion. Therefore, controlling the proliferation of VSMCs is fundamental in preventing post-vascular injury stenosis. In recent years, multiple studies and drugs have attempted to improve vascular injury-related stenosis by regulating VSMC proliferation, but the therapeutic efficacy has been unsatisfactory. Therefore, further investigation of the molecular mechanisms underlying vascular injury-induced proliferation is essential to find potential therapeutic targets.

To identify potential regulatory factors and therapeutic targets for vascular injury-induced neointimal formation, we conducted comprehensive biological analyses, including transcriptome sequencing of injured mouse carotid artery tissues (GSE40637) and VSMCs induced with PDGF-BB. Subsequently, we identified potential key genes that could play a pivotal role in the context of vascular injury. To further refine our selection, we performed RT-PCR to assess the expression levels of these key genes in VSMCs treated with PDGF-BB. From this analysis, KIF11 (kinesin family member 11) may emerge as a noteworthy candidate implicated in the development of vascular restenosis following injury.

KIF11, a kinesin motor protein family member, functions as a molecular motor that moves unidirectionally along microtubule tracks (Chen and Hancock, 2015; Simunić and Tolić, 2016; Venuto et al., 2020). It plays a crucial role in the formation of the bipolar spindle apparatus, a pivotal process in mitosis (Rath and Kozielski, 2012). Furthermore, literature reports suggest that KIF11 is implicated in promoting protein secretion and cellular transport (Rath and Kozielski, 2012). Inhibiting the expression and activity of KIF11 can lead to cell cycle arrest, reduced proliferative capacity, and the initiation of apoptosis (Liu et al., 2017; Gu et al., 2022). Therefore, KIF11 plays a significant role in regulating cell survival and proliferation. However, the mechanisms through which KIF11 regulates in VSMCs proliferation and vascular neointimal hyperplasia remain unclear.

This study used a guidewire-induced carotid artery injury model to evaluate the function of inhibiting KIF11 activity on VSMCs proliferation and neointima formation following vascular injury. Our findings demonstrated that inhibiting KIF11 activity led to cell cycle arrest and reduced the proliferative capacity in VSMCs, thereby suppressing neointima formation after vascular injury. *In vitro* experiments revealed that suppressing KIF11 expression and using the specific KIF11 inhibitor K858 could partially reverse the PDGF-BB-induced effects on VSMCs cell cycle progression and proliferation. These data suggest that KIF11 mediates VSMCs proliferation and vascular neointima formation, indicating that inhibiting KIF11 expression and activity in VSMCs could be a novel strategy to counteract neointimal formation following vascular injury.

## 2 Materials and methods

### 2.1 Animal

Eight-week-old male C57 mice were procured from Shanghai Vital River Laboratory Animal Technology Co. (Beijing, China). The mice were housed in a specific pathogen-free (SPF) environment. The animal experimental protocol was approved by the Animal Care Committee of Tongji Medical College, Huazhong University of Science and Technology. The IACUC approval number is [2023] 3496.

### 2.2 Vascular injury

The method for inducing carotid artery injury in mice was performed following the procedure described by Tripathi M (Tripathi et al., 2022). Mice were anesthetized with isoflurane (2.5%–3%). A midline neck incision was made after inducing anesthesia, and the skin was carefully dissected layer by layer. The carotid artery, internal carotid artery, and external carotid artery were bluntly separated. Temporary control of blood flow in the carotid artery and internal carotid artery was achieved using vascular clamps. The external carotid artery was ligated at the proximal end using an 11–0 nylon suture, followed by a slight incision at the distal end of the external carotid artery at a 45° angle using microscissors. A 0.38 mm diameter metal guidewire was inserted through the incision into the external carotid artery and passed through to the carotid artery. The guidewire was gently moved within the carotid artery approximately 10 times. After removing the guidewire, the external carotid artery was ligated at the proximal end, and blood flow was restored in the internal carotid artery and carotid artery. The neck wound was sutured using 6–0 nylon thread. The mice were randomly divided into three groups, including the injury group and inhibitor group, and were administered either DMSO or K858 (50 mg/kg, Selleck) every other day for 2 weeks post-injury. After 2 weeks, mice were euthanized with an overdose of pentobarbital sodium (180 mg/kg) via intraperitoneal injection. The carotid artery segments from the injured area were harvested, and the vessels were perfused *in situ* with ice-cold sterile physiological saline. The specimens were used for histological and immunohistochemical analysis.

### 2.3 Immunohistochemistry and immunofluorescence

Following tissue excision, specimens were fixed in 4% paraformaldehyde and subsequently embedded in paraffin blocks. Subsequently, 5 μm-thick sections were deparaffinized in xylene and then subjected to ethanol washes at varying concentrations. The sections were then subjected to Hematoxylin and Eosin (HE) staining, Elastica van Gieson (EVG) staining, and immunohistochemical staining for KIF11. The intima-to-media ratio and intimal area were calculated using established statistical methods, as previously described in our prior research (Zheng et al., 2022). EVG staining was employed to distinguish between the intimal and medial regions of blood vessels, and the areas of media, new intima, and intimal side were quantified using ImageJ (software, Maryland, United States). Digital images were captured using an inverted microscope (Olympus, Tokyo, Japan).

### 2.4 Cell culture and treatment

Primary VSMC cells were extracted through the tissue explant method (Langenickel et al., 2008). The thoracic aorta from mice was dissected and cut into regular tissue blocks. These tissue blocks were attached to the culture surface, incubated in DMEM/DF12 medium with 20% FBS (Life Technologies, Grand Island, United States) and 20 ng/mL PDGF-BB (Peprotech, New Jersey, United States), and cultured under conditions of 37°C and 5% CO2. Upon primary cell outgrowth, the medium was replaced with DMEM/DF12 medium containing 10% FBS for continued cultivation.

Recombinant plasmids for KIF11 (KIF11-Plvx-AcGFP1-N1) were constructed (Genecreate, Wuhan, China), as well as siRNA. Transfection was carried out when the cell density reached approximately 50%–60%. The siRNA sequences for KIF11 were 5′-CCU​UGA​UGA​AUG​CUU​ACU​CUA-3′ and 5′-UAG​AGU​AAG​CAU​UCA​UCA​AGG-3′, while the control group was transfected with siRNA sequences 5′-UUC​UCC​GAA​CGU​GUC​ACG​U-3′ and 5′-ACG​UGA​CAC​GUU​CGG​AGA​A-3′ (Genecreate, Wuhan, China). Specific KIF11 inhibitor K858 was chosen, based on the KIF11-specific inhibitor reported by Nakai R and Nicolai A (Nakai et al., 2009; Nicolai et al., 2022). For pretreatment, cells were starved in a medium containing 0.2% BSA for 24 h before being stimulated with PDGF-BB for an additional 24 h.

### 2.5 Quantitative RT-PCR

Total RNA was isolated using a TRIzol reagent (Vazyme, Nanjing, China). Reverse transcription was performed separately using the iCycler real-time fluorescence quantitative PCR detection system (Bio-Rad) and Supermix (R122-01, Vazyme). Quantitative real-time reverse transcription-PCR (RT-PCR) was carried out using the SYBR Green reagent kit (Q111-02, Vazyme). The 2^−ΔΔCT^ method was employed with Actin as the reference gene. The primer sequences were as follows: KIF11, 5′-AGT​GCG​AAA​CAA​AAG​GCC​AT-3' and 5′-TGT​GCT​GCT​AAC​GAT​TGC​TC-3'; Actin, 5′-GTG​CTA​TGT​TGC​TCT​AGA​CTT​CG-3' and 5′-ATG​CCA​CAG​GAT​TCC​ATA​CC-3'.

### 2.6 Western blotting

Protein extraction was carried out using standard methods with RIPA lysis buffer. Briefly, proteins were separated using SDS-PAGE gels and subsequently transferred to a nitrocellulose (NC) membrane. After blocking with skim milk for 1 h, the respective primary antibodies were incubated with the membrane. Following overnight incubation at 4°C with the primary antibodies, the membrane was washed three times with TBST and then incubated with the corresponding secondary antibodies for 1 h. Bands of different proteins were visualized and recorded using the ChemiDoc imaging system (Bio-Rad). The antibodies used in this study were as follows: Cyclin D1 (ab40754,1:1,000, Abcam), CDK4 (ab7955, 1:1,000, Abcam), Cyclin E1 (ab71535, 1:2,000, Abcam), CDK2 (ab32147, 1:1,000, Abcam), PCNA (rabbit 1:100, Abcam), CDK6 (14052-1-AP, 1:1,000, Proteintech), β-tubulin (11224-1-AP, 1:5,000, Proteintech), AKT (60203-2-Ig, 1:1,000, Proteintech), p-AKT (66444-1-Ig, 1:500, Proteintech). β-tubulin was used as a loading control.

### 2.7 Cell cycle analysis

Flow cytometry was employed to determine the distribution of cells in different phases of the cell cycle. Following siRNA transfection and treatment with the KIF11 inhibitor, VSMC were stimulated with PDGF-BB (20 ng/mL) for 24 h. Cells were collected, washed twice with PBS, and then fixed in 70% ethanol overnight. Before analysis, cells were stained with propidium iodide (C1052, Beyotime) for 30 min. Sample analysis was conducted using a FACS machine (Becton, Dickinson and Company, New Jersey, United States).

### 2.8 Cell proliferation assay

VSMC Cell Sequencing Analysis: VSMC were serum-starved in a medium containing 0.2% BSA for 24 h after reaching 80% confluence. Subsequently, they were induced with 20 ng/mL of PDGF-BB for another 24 h before sample collection for cell transcriptome sequencing. Genes with Log2FC > 2 and *p*-value<0.05 were considered as differentially expressed genes (DEGs). DEGs from cell transcriptome sequencing were intersected with DEGs from GSE40637, followed by protein-protein interaction analysis using the STRING database. Subsequently, network analysis was performed using the CytoNCA plugin in Cytoscape, focusing on nodes with a Betweenness Centrality (BC) score greater than 10 for further investigation.

### 2.9 CCK-8 analysis

VSMC proliferation was assessed using the CCK-8 assay. In brief, 2 × 10^3 VSMC cells were seeded in each well of a 96-well plate and stimulated with PDGF-BB (20 ng/mL) for the specified duration following transfection or inhibitor treatment. Subsequently, cells were incubated with CCK-8 solution in the dark for 2 h. The absorbance of the samples was measured at 450 nm using a microplate reader (Thermo, Massachusetts, United States).

### 2.10 EdU incorporation assay

Pre-treated VSMC cells were incubated in the dark for 2 h with serum-free culture medium containing 20 μM EDU working solution after a partial medium change. After labeling, cells were fixed in 4% paraformaldehyde. Subsequent steps were carried out according to the instructions provided in the assay kit. Following staining, cell nuclei were counterstained with Hoechst for 5 min.

### 2.11 Statistical analysis

At least three replications of each test were performed for statistical analysis. The Shapiro-Wilk test initially assessed the normality of the data. For data that did not conform to a normal distribution, the Kruskal–Wallis test was used. In cases where the data conformed to a normal distribution, homogeneity of variance was assessed using the Lexeme test. Brown-Forsythe and Tamhane’s T2 tests were performed for data exhibiting heteroscedasticity. Statistical methods used included one-way and two-way analyses of variance (ANOVAs) and Student’s t-tests. Each experiment was conducted with a minimum of three replicates, and the data are presented as mean ± standard deviation (SD). Data were visualized and statistically analyzed using prism graphpad 8 (GraphPad SoftwareInc., San Diego, CA, United States) for processing. *p* < 0.05 was considered to be a significant difference.

## 3 Results

### 3.1 KIF11 is upregulated in PDGF-BB-induced VSMCs and injured carotid artery in mice

**FIGURE 1:**
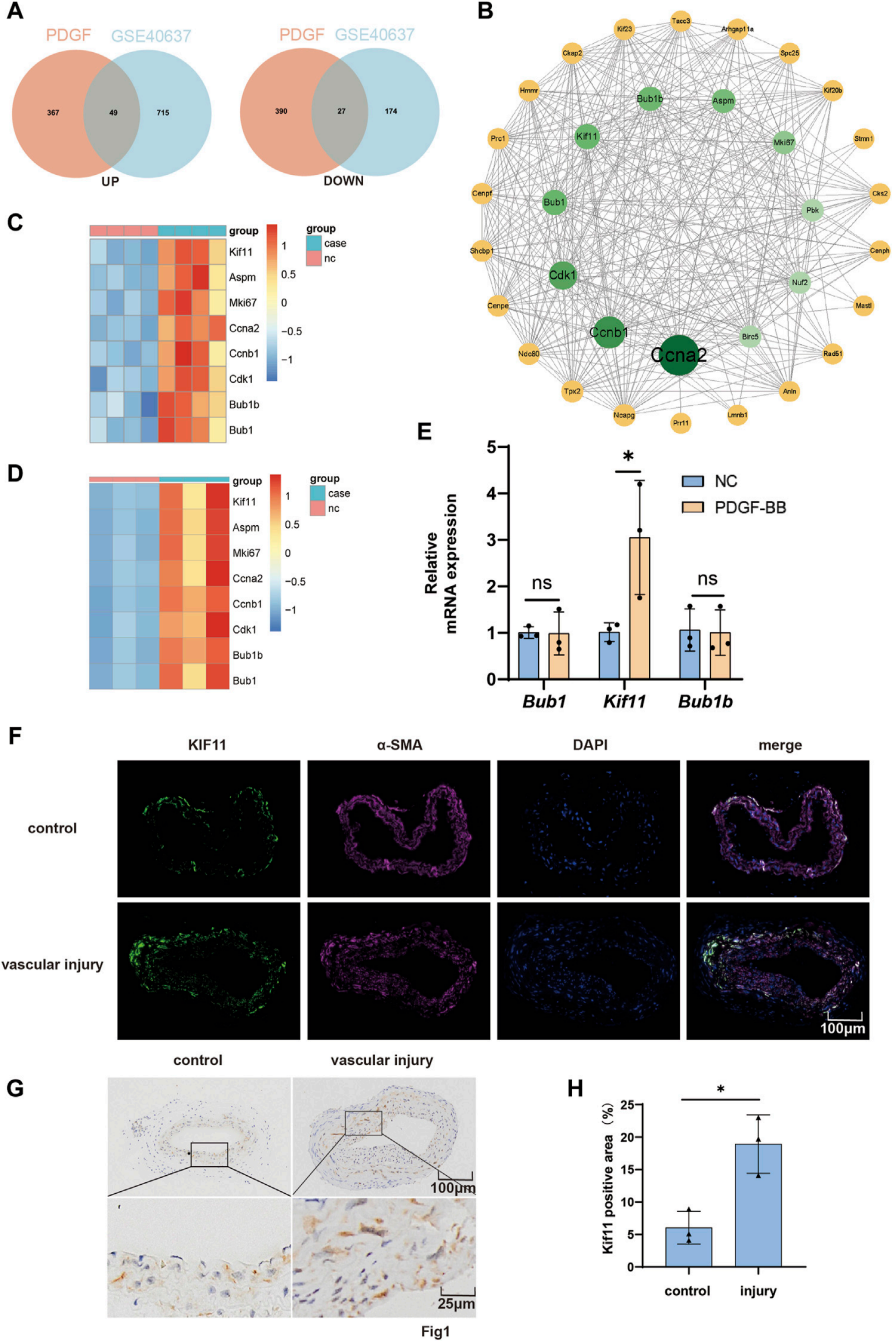
KIF11 is upregulated in PDGF-BB-induced VSMCs and injured carotid artery in mice. **(A)** VSMCs were treated with PDGF-BB (20 ng/mL) for 24 h, and then subjected to cell transcriptome sequencing. The differentially expressed genes from the cell transcriptome sequencing results, including both upregulated and downregulated genes, were compared with the differentially expressed genes from the GEO database GSE40637. **(B)** The 49 overlapping upregulated genes and the 27 overlapping downregulated genes were used to construct a protein-protein interaction network in the STRING database in order to identify hub genes. **(C,D)** Create a protein-protein interaction network using Cytoscape software. Network analysis was performed using the CytoNCA plugin in Cytoscape, focusing on nodes with a Betweenness Centrality (BC) score greater than 10 for further investigation. Generate a heatmap of the genes with a score greater than 10 from the GSE40637 dataset and the cell transcriptome sequencing data. **(E)** After treating VSMCs with PDGF-BB (20 ng/mL) for 24 h, assess the expression levels of the top three hub genes (Bub1, Kif11, and Bub1b) from the protein-protein interaction network using RT-PCR. Normalize mRNA expression to β-ACTIN, N = 3 *p < 0.05, **p < 0.01, ***p < 0.001. **(F)** Conduct immunostaining of representative cross-sections of the injured carotid artery to visualize KIF11 (green) and α-SMA (red). N = 3 *p < 0.05, **p < 0.01, ***p < 0.001. **(G,H)** Perform immunohistochemical staining for KIF11 on cross-sections of representative injured carotid artery segments. The bar chart **(H)** should display the average percentage of IHC-positive cells in the intimal and neointimal regions. N = 3 *p < 0.05, **p < 0.01, ***p < 0.001.

To identify key genes responsible for neointima formation following vascular injury and potential therapeutic targets, VSMCs were subjected to serum starvation and stimulated with PDGF-BB for 24 h before sample collection for cell sequencing. Cross-reference the upregulated differentially expressed genes identified in cellular sequencing with those in the upregulated differential genes from the GEO database GSE40637. Similarly, perform an intersection analysis for the downregulated genes. (Figure 1A). After that, a protein-protein interaction network was constructed with 49 upregulated intersected genes and 27 downregulated intersected genes to identify hub genes (Figure 1B). Heatmaps of genes with interaction network scores greater than 10 in their respective datasets were plotted (Figures 1C, D).

Given that ccna2, ccnb1, and cdk1 are classical cell cycle-related proteins and have been confirmed to play crucial roles in neointimal formation following vascular injury by regulating cell cycle progression (Wei et al., 1997; Braun-Dullaeus et al., 1999; Silvestre-Roig et al., 2013), we measured the mRNA expression levels of other top-ranking genes (Bub1, KIF11, and Bub1b) in the protein interaction network after PDGF-BB treatment for 24 h using RT-qPCR. Bub1 and Bub1b are localized to the kinetochore and play a crucial role in inhibiting the anaphase-promoting complex/cyclosome (APC/C). This inhibition delays the onset of anaphase, ensuring accurate chromosome segregation. However, the results only showed a significant upregulation of KIF11 mRNA expression by PDGF-BB (Figure 1E). Furthermore, tissue immunofluorescence demonstrated significant co-localization of KIF11 and α-SMA, with increased KIF11 expression in the injured side of the blood vessels (Figure 1F). Immunohistochemistry also confirmed the upregulation of KIF11 expression in the injured artery (Figure 1G–H). Then, we induced VSMCs with varying concentrations of PDGF-BB and explored whether KIF11 is regulated by PDGF-BB through RT-qPCR and Western blotting. As expected, the results from both Rt-PCR and Western blotting indicated that PDGF-BB upregulates the mRNA and protein expression levels of KIF11 in VSMCs, with the strongest effect observed at 20 ng/mL (Supplementary Figures S1A–C). Furthermore, under the influence of 20 ng/mL PDGF-BB, KIF11 expression was significantly upregulated after 24 h (Supplementary Figures S1D–F). The above results indicate an upregulation of KIF11 expression in arterial smooth muscle cells and PDGF-BB-induced VSMCs.

### 3.2 KIF11 mediates PDGF-BB-induced cell cycle progression of VSMCs

**FIGURE 2:**
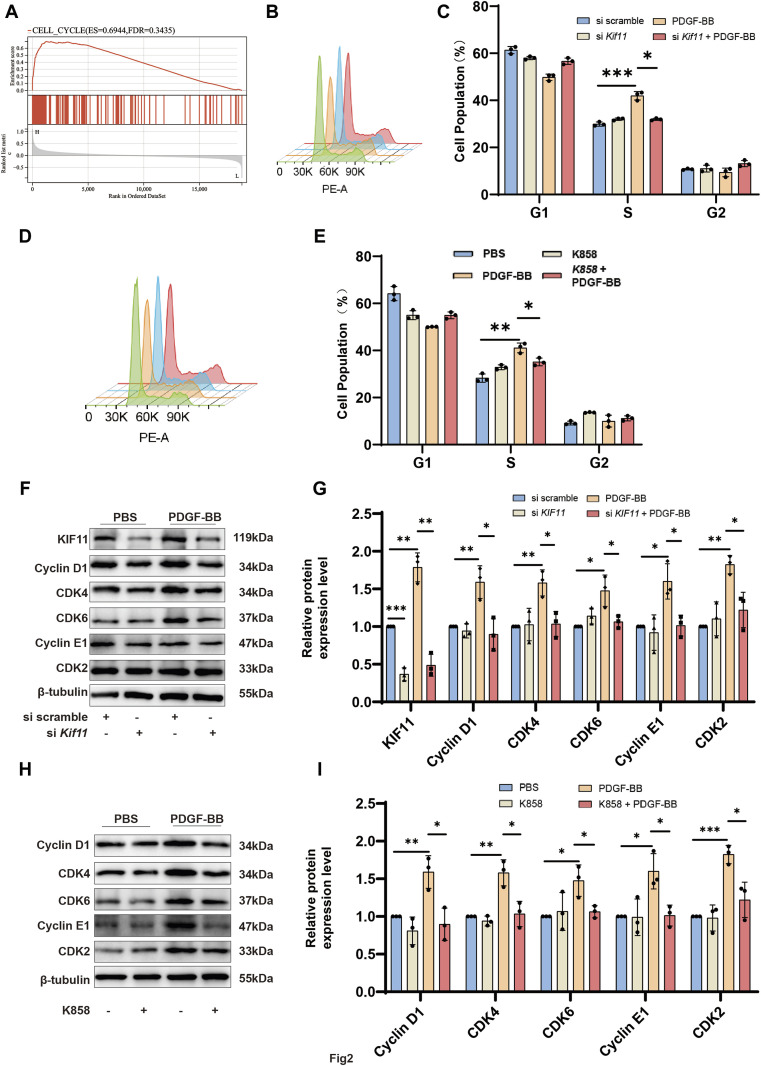
KIF11 mediates PDGF-BB-Induced cell cycle progression of VSMCs. **(A)** Perform Gene Set Enrichment Analysis (GSEA) for KIF11 in GSE40637. Divide the samples into high-expression group (≥50%) and a low-expression group (<50%) based on gene expression profiles and phenotypic grouping. Set the minimum gene set to 5, and the maximum gene set to 5,000, perform 1,000 permutations, and consider it statistically significant if it has a *p*-value of <0.05 and FDR of <0.25. **(B,C)** VSMCs in the control group and si*Kif11* group were treated with PDGF-BB (20 ng/mL) for 24 h, and cell cycle progression was analyzed using flow cytometry. Bar chart **(C)** displays the proportion of cells in different cell cycle phases for the control and si*Kif11* groups. N = 3 *p < 0.05, **p < 0.01, ***p < 0.001. **(D,E)** VSMCs in the control group and K858 group were treated with PDGF-BB (20 ng/mL) for 24 h, and cell cycle progression was analyzed using flow cytometry. Bar chart **(E)** displays the proportion of cells in different cell cycle phases for the control and K858 groups. N = 3 *p < 0.05, **p < 0.01, ***p < 0.001. **(F,G)** After treating si scramble group and si*Kif11* group VSMCs with PDGF-BB for 24 h, the protein expression levels of cell cycle-related proteins, including Cyclin D1, CDK4, CDK6, Cyclin E1, and CDK2, were quantified using Western blot analysis. Bars **(G)** indicate the relative expression levels of the proteins. N = 3 *p < 0.05, **p < 0.01, ***p < 0.001. **(H,I)** After treating VSMCs with PDGF-BB (20 ng/mL) and K858 for 24 h, the protein expression levels of cell cycle-related proteins, including Cyclin D1, CDK4, CDK6, Cyclin E1, and CDK2, were quantified using Western blot analysis. Bars **(I)** indicate the relative expression levels of the proteins. N = 3 *p < 0.05, **p < 0.01, ***p < 0.001.

KIF11 has been confirmed to play a crucial role in regulating the cell cycle and proliferation (Rath and Kozielski, 2012; Liu et al., 2017). However, its function in VSMCs has not been cleared. To investigate the mechanism of KIF11 in VSMCs, we conducted a KIF11 single-gene Gene Set Enrichment Analysis (GSEA) using the GSE40637 dataset. The results revealed that KIF11 positively regulates cell cycle progression (Figure 2A). Compared to the control group, PDGF-BB stimulated VSMCs to significantly increase the S-phase cell population while decreasing the G0/G1-phase cell population. However, knocking down KIF11 disrupted this trend (Figures 2B, C). Additionally, inhibiting KIF11’s activity with the KIF11-specific inhibitor, K858 (1 μM), also blocked the effect of PDGF-BB on promoting cell cycle progression in VSMC (Figures 2D, E). These findings provide evidence that KIF11 plays a critical role in PDGF-BB-induced VSMC cell cycle progression.

Cell cycle progression from G0/G1 to the S phase depends on the precise regulation of the Cyclin D1/CDK4, CDK6, and Cyclin E1/CDK2 systems. To explore the potential mechanism by which KIF11 regulates cell cycle progression, we measured the expression levels of cell cycle-related proteins using Western blotting. The results indicated that compared to the control group, PDGF-BB treatment in VSMCs led to a significant upregulation of Cyclin D1, CDK4, CDK6, Cyclin E1, and CDK2. However, knocking down KIF11 inhibited the upregulation of these proteins induced by PDGF-BB (Figures 2F, G). Similarly, using K858 to inhibit KIF11’s activity also suppressed PDGF-BB’s effect on promoting cell cycle progression in VSMC (Figures 2H, I). ANNEXIN-V flow cytometry results showed that inhibition of both KIF11 expression and activity did not induce significant apoptosis (Supplementary Figures S2A, B). These results confirm that KIF11 mediates PDGF-BB-induced VSMC cell cycle progression.

### 3.3 KIF11 is required for VSMCs proliferation

**FIGURE 3:**
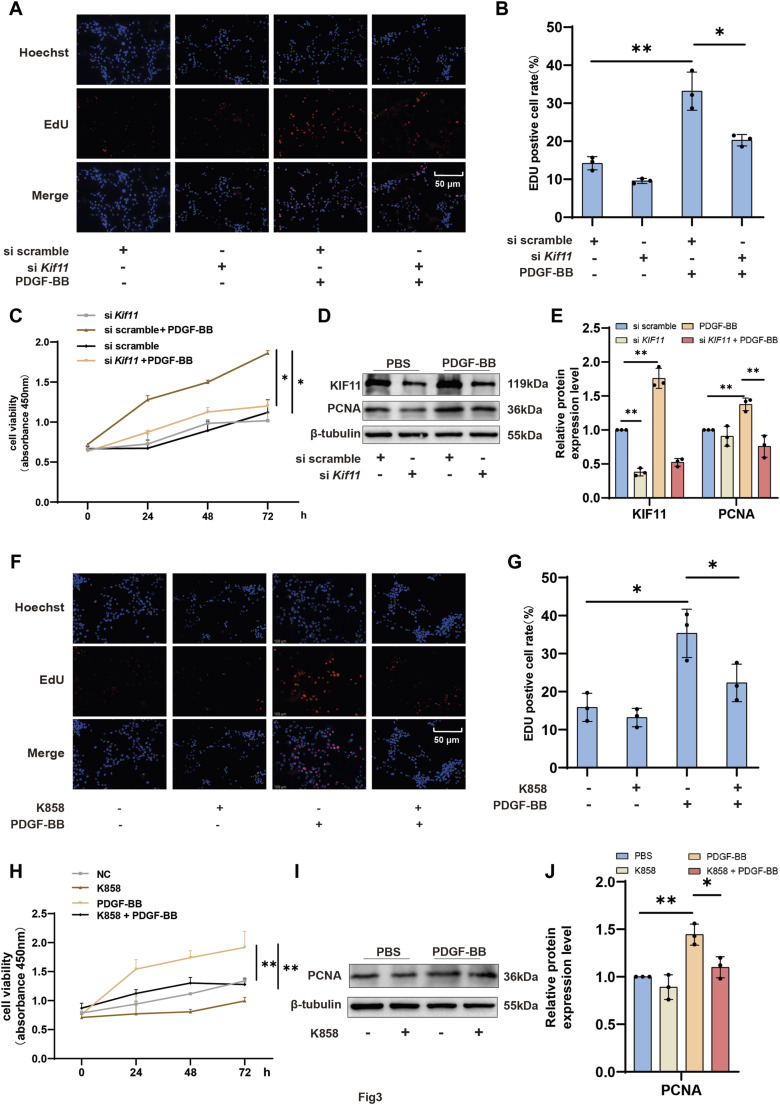
KIF11 is required for VSMCs proliferation **(A,B)** After treating the control group and K858 group VSMCs with PDGF-BB for 24 h, the proliferative capacity of VSMCs was assessed using EDU labeling. Panel **(B)** presents the percentage of EDU-positive cells in each group. N = 3 *p < 0.05, **p < 0.01, ***p < 0.001. **(C)** Following a 24-h treatment of VSMCs with PDGF-BB in the si scramble group and si*Kif11* group, the proliferative capacity of VSMCs was assessed using CCK-8. **(D,E)** After treating si scramble group and si*Kif11* group VSMCs with PDGF-BB for 24 h, the protein expression levels of PCNA were quantified using Western blotting. Bars **(E)** indicate the relative expression levels of the proteins. N = 3 *p < 0.05, **p < 0.01, ***p < 0.001. **(F,G)** Following a 24-h treatment of VSMCs with PDGF-BB in the control group and K858 group, the proliferative capacity of VSMCs was assessed using CCK-8. Bar graph **(G)** displays the percentage of EDU-positive cells in each group. **(H)** After treating the control group and the K858 group of VSMCs with PDGF-BB for 24 h, their proliferative capacity was assessed using the CCK-8 assay. **(I,J)** After treating the control group and the K858 group of VSMCs with PDGF-BB for 24 h, the protein expression levels of PCNA were quantified using Western blot analysis *p < 0.05, **p < 0.01, ***p < 0.001. Bars **(J)** indicate the relative expression levels of the proteins. N = 3 *p < 0.05, **p < 0.01, ***p < 0.001.

To investigate the relationship between KIF11 and VSMCs proliferation, we assessed VSMCs proliferation using EDU and CCK-8 assays. As expected, the results were consistent with our expectations, showing that knocking down KIF11 partially reversed the pro-proliferative effect of PDGF-BB on VSMC (Figures 3A–C). Furthermore, we verified VSMCs proliferation by detecting the protein expression level of PCNA, which showed that PDGF-BB treatment led to a significant upregulation of PCNA protein expression compared to the control group. However, knocking down KIF11 partially inhibited the upregulation of PCNA by PDGF-BB (Figures 3D, E). Consistent with the KIF11 knockdown results, the use of the KIF11-specific inhibitor K858 also partially reversed the pro-proliferative effect of PDGF-BB on VSMC (Figures 3F–H). K858 also reversed the upregulation of PCNA by PDGF-BB (Figures 3I, J). These findings indicate that KIF11 mediates PDGF-BB’s regulation of VSMC proliferation. PDGF-BB promotes VSMC cell cycle progression and proliferation by upregulating both the expression and activity of KIF11.

### 3.4 The PI3K/AKT pathway is responsible for promoting the expression of KIF11 and cell proliferation

**FIGURE 4:**
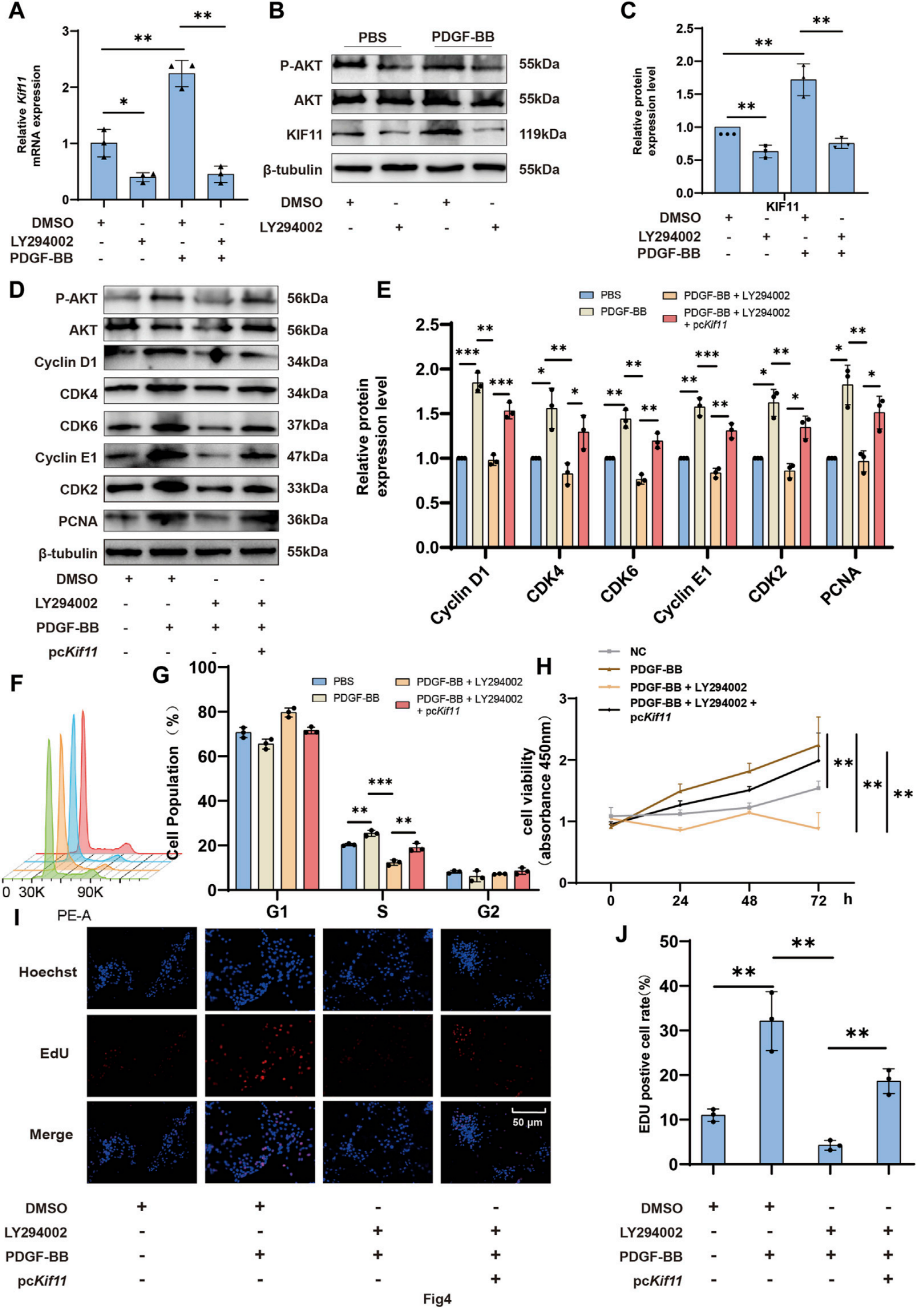
The PI3K/AKT pathway is responsible for promoting the expression of KIF11 and cell proliferation. **(A–C)** VSMCs in the control group and LY294002 group were starved for 24 h using a serum-free medium containing 0.2% bovine serum albumin (BSA). They were then treated with PDGF-BB (20 ng/mL) for an additional 24 h. The expression levels of KIF11 mRNA and protein were measured using rt-PCR and Western blot. Bars **(C)** indicate the relative expression levels of the proteins. N = 3 *p < 0.05, **p < 0.01, ***p < 0.001. **(D,E)** VSMCs in the control and pc*Kif11* groups were serum-starved for 24 h with 0.2% BSA medium. They were then exposed to PDGF-BB and LY294002 for an additional 24 h. Cell cycle progression was assessed using flow cytometry, and the bar chart **(G)** shows the cell cycle phase distribution in each group. N = 3. *p < 0.05, **p < 0.01, ***p < 0.001. **(F–G)** Following a 24-h treatment of pc*Kif11* group VSMCs with PDGF-BB and LY294002, protein expression levels of cell cycle-related proteins were assessed using Western blotting. Bars **(I)** indicate the relative expression levels of the proteins. N = 3 *p < 0.05, **p < 0.01, ***p < 0.001. **(H)** After a 24-h treatment of pc*Kif11* group VSMCs with PDGF-BB and LY294002, cell proliferation capacity was assessed using the CCK-8 assay. **(I,J)** After a 24-h treatment of pc*Kif11* group VSMCs with PDGF-BB and LY294002, cell proliferation capacity was assessed using the EDU assay. Bar graph **(J)** displays the percentage of EDU-positive cells in each group. N = 3 *p < 0.05, **p < 0.01, ***p < 0.001.

Consistent with others, our previous studies have shown that PDGF-BB regulates VSMCs proliferation through the PI3K/AKT pathway (Kaplan-Albuquerque et al., 2003; Dong et al., 2018; Yu et al., 2018; Yu et al., 2019). There is also evidence suggesting that the PI3K/AKT pathway plays a crucial role in KIF11’s promotion of cell cycle progression and proliferation (Wei et al., 2021; Wei et al., 2022; Zhu et al., 2023). Therefore, we decided to investigate whether PDGF-BB upregulates KIF11 expression through the PI3K/AKT pathway. RT-qPCR and Western blot results showed that, compared to the control group, treatment with the specific PI3K/AKT inhibitor LY294002 significantly inhibited AKT activity and led to a moderate decrease in KIF11 expression. LY294002 also markedly blocked PDGF-BB’s pro-expression effect on KIF11 (Figures 4A–C), indicating that PDGF-BB may regulate KIF11 expression through the PI3K/AKT pathway. In addition, the use of rapamycin likewise partially inhibited KIF11 expression (Supplementary Figures S2C, D). In comparison to control cells, LY294002 significantly inhibited the promotion of VSMC cell cycle progression by PDGF-BB, resulting in a significant reduction in the S-phase cell population and an increase in the G0/G1-phase cell population. However, overexpression of KIF11 partially alleviated the inhibitory effect of LY294002 on PDGF-BB’s biological function (Figures 4D, E). To further explore the underlying mechanisms, we assessed the expression levels of cell cycle-related proteins and KIF11 through Western blot analysis. The results demonstrated that LY294002 significantly inhibited PDGF-BB’s pro-expression effect on KIF11, subsequently affecting the pro-expression effects of PDGF-BB on Cyclin D1, CDK4, CDK6, Cyclin E1, CDK2, and other cell cycle-related proteins, as well as PCNA. However, the inhibitory effect of LY294002 on PDGF-BB’s biological function was partially reversed in the pcKif11, LY294002, and PDGF-BB co-treated group. In this group, the expression levels of Cyclin D1, CDK4, CDK6, Cyclin E1, CDK2, and PCNA were upregulated compared to the LY294002 and PDGF-BB co-treated group. This suggests that PDGF-BB regulates KIF11 expression through the PI3K/AKT pathway, thereby influencing VSMC cell cycle progression (Figures 4F, G). Interestingly, we found that the ratio of p-AKT/AKT was upregulated in the pcKIF11, LY294002, and PDGF-BB co-treated group compared to the LY294002 and PDGF-BB co-treated group, indicating that PDGF-BB can regulate KIF11 expression through the PI3K/AKT pathway, and KIF11 expression may further activate the PI3K/AKT pathway, forming a positive feedback loop. Similarly, results from CCK-8 and EDU assays showed that cell proliferation in the pcKif11, LY294002, and PDGF-BB co-treated group was restored compared to the LY294002 and PDGF-BB co-treated group (Figures 4H–J). This suggests that PDGF-BB promotes cell proliferation by upregulating KIF11 expression through the PI3K/AKT pathway.

### 3.5 Inhibition of KIF11 attenuates neointimal formation by suppressing VSMCs proliferation

**FIGURE 5:**
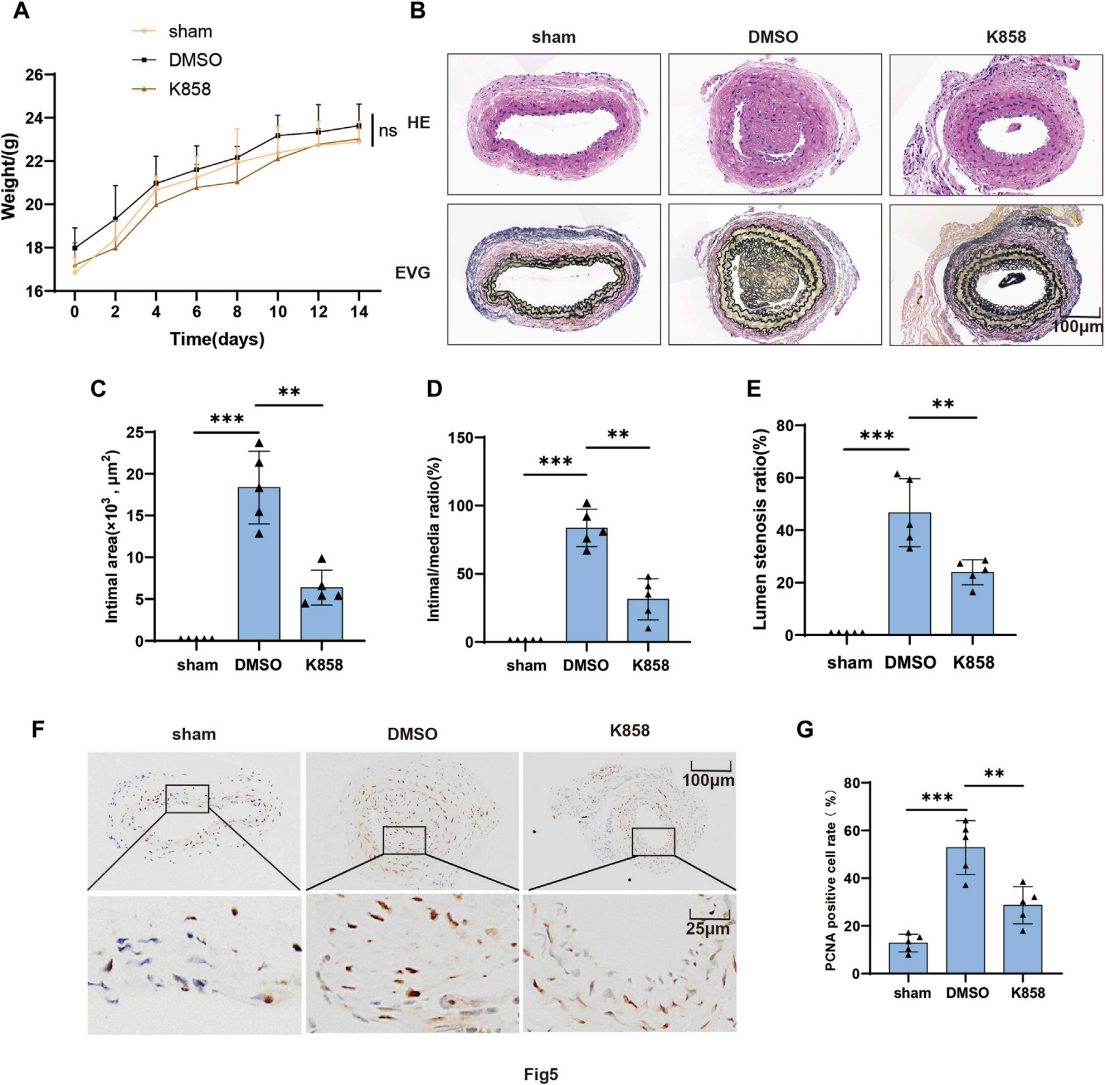
Inhibition of KIF11 attenuates neointimal formation by suppressing VSMCs proliferation. **(A)** Weights of control, injury, and inhibitor groups of mice were measured every 2 days following vascular injury, and the results were plotted as a line graph. **(B–E)** Representative cross-sections of injured segments of carotid arteries from control, injury, and inhibitor groups of mice were subjected to HE staining and EVG staining. The bar graphs in **(C–E)** display the intimal area, intima/media ratio, and Lumen stenosis ratio. N = 5, *p < 0.05, **p < 0.01, ***p < 0.001. **(F,G)** Representative cross-sections of injured segments of carotid arteries from the injury and inhibitor groups of mice were subjected to PCNA immunostaining. The bar graphs **(G)** show the average percentage of IHC-positive cells in the intimal and neointimal regions, N = 5 *p < 0.05, **p < 0.01, ***p < 0.001.

The proliferation of VSMCs is closely associated with the neointima formation. Our *in vitro* experiments have demonstrated that the progression of the VSMCs cell cycle and proliferation is regulated by the expression and activity of KIF11. To further investigate the role of KIF11 in the development of neointima formation following carotid artery injury, we administered KIF11-specific inhibitor K858 (50 mg/kg) via intraperitoneal injection in a mice model of carotid artery injury. Observations of weight for the injury group and inhibitor group indicated that the KIF11 inhibitor did not affect the normal growth of the mice, with no significant differences in body weight between the two groups (Figure 5A). A comparison of the H&E and EVG staining of vascular specimens in the two groups revealed that, in comparison to the injury group, the inhibitor group exhibited a significant reduction in intimal area, intima/media ratio, and Lumen stenosis ratio within the injured segment of blood vessels (Figures 5B–E). Subsequently, we conducted an immunohistochemical analysis to assess the impact of inhibiting KIF11 activity on neointima formation following vascular injury. Evaluation of VSMC proliferative capacity by examining the PCNA as a marker revealed a significant decrease in the proportion of PCNA-positive VSMC in the injured blood vessel segments following *in vivo* inhibition of KIF11 activity, compared to the control group (Figures 5F, G). These results suggest that KIF11 activity plays a crucial role in intimal hyperplasia after vascular injury, and inhibiting KIF11 activity could serve as a potential therapeutic approach to prevent and suppress neointimal formation following vascular injury.

## 4 Discussion

Vascular injury stands as a common and significant cause of luminal narrowing in various clinical procedures, such as angioplasty and stenting. Vascular injury disrupts the integrity of the vascular endothelium, resulting in platelet deposition and leukocyte aggregation in the affected area. This process is accompanied by the release of various inflammatory and growth factors. The secreted inflammatory factors and growth factors promote smooth muscle cell proliferation leading to neointima formation and causing vascular luminal stenosis. The migration and proliferation of VSMCs play a crucial role in neointima formation. However, the cell cycle regulatory mechanism underlying the injury-induced proliferation of VSMCs remains unclear. Under normal conditions, the proliferation of VSMCs is limited due to their sufficient differentiation. However, when VSMCs are stimulated by inflammatory factors or other stimuli, they re-enter the cell cycle to resume their ability to proliferate (Frismantiene et al., 2018). Numerous findings suggest that therapeutic relief of neointimal formation can be achieved by early inhibition of the cycle progression of VSMCs. The cell cycle is regulated by cytokines, CDKs, and regulatory cyclin subunits, which form the cell cycle signaling pathway and play a central role in cell proliferation and migration as well as growth and metabolism. While cell cycle progression is orchestrated by CDKs and their regulatory cyclin subunits, the actual initiators of the cell cycle are multiple signaling pathways activated by cytokines (Smith et al., 2019). The PI3K/AKT pathway serves as a pivotal mediator in various critical aspects of stenosis following vascular injury. It is a key signaling pathway orchestrating the proliferation and migration of VSMCs induced by a variety of growth factors or cytokines (e.g., PDGF, TNF-α, HMGB1, and IL-6). This pathway plays a crucial role in regulating VSMCs growth, proliferation, mRNA and protein processing, and synthesis, making it a prominent focus in current research (Sheu et al., 2005; Yue et al., 2019). Meanwhile, studies have shown that inhibition of the activity of the PI3K/AKT pathway induces cycle arrest in VSMCs and inhibits neointima formation (Caglayan et al., 2011; Li et al., 2015). However, the mechanism of how PI3K/AKT mediates vascular endothelial proliferation has not been clarified.

We employed transcriptomic analysis to further understand the proliferation of VSMCs and the underlying mechanisms of neointima formation. In this study, we identified the target gene KIF11 by analyzing the results of tissue transcriptome sequencing of injured segmental blood vessels in mice and cellular transcriptome sequencing after PDGF-BB stimulation of VSMCs *in vitro*. KIF11, a member of the kinesin protein family, plays a key role in regulating mitosis by influencing spindle formation and intracellular material transport. It has been previously established that KIF11 is associated with adverse outcomes in various diseases, such as gallbladder and pancreatic cancer, primarily due to its ability to modulate cell proliferation and vitality (Wei et al., 2021; Gu et al., 2022; Zhu et al., 2023). Our results reveal that KIF11 also plays a crucial role in vascular intimal hyperplasia following vascular injury for the first time. By creating an animal model using guidewire-induced injury in mouse carotid arteries, we employed immunohistochemistry to demonstrate the upregulation of KIF11 expression in response to vascular injury. Histological staining with H&E and EVG further confirmed that inhibiting KIF11 activity could effectively suppress neointimal formation after vascular injury. To further elucidate the mechanisms and functions of KIF11 in neointima formation, we conducted *in vitro* experiments using PDGF-BB to stimulate VSMC. The results indicated that PDGF-BB can enhance VSMC cell cycle progression and proliferation by upregulating KIF11 expression and activity through the PI3K/AKT pathway. These *in vitro* findings are consistent with the results of our animal experiments, which showed that reducing KIF11 expression and activity partially counteracted the pro-proliferative and cell cycle-promoting effects of PDGF-BB on VSMC. Collectively, these data provide the first evidence that PDGF-BB promotes VSMC proliferation through the upregulation and activation of KIF11.

However, in this study, we did not investigate the precise mechanisms through which KIF11 facilitates cell cycle progression and promotes proliferation. Additionally, results from Wei D and others have suggested that KIF11 exerts its biological effects by activating the ERBB2/PI3K/AKT pathway (Wei et al., 2021). In contrast, our experimental findings demonstrate that PDGF-BB upregulates KIF11 expression and activity through the PI3K/AKT pathway in VSMC. These discrepancies may arise from differences between cell types. Nevertheless, further research is required to explore the specific mechanisms by which KIF11 positively regulates the PI3K/AKT pathway. Furthermore, in animal experiments, we reduced KIF11 activity by administering K858, a KIF11-specific inhibitor, via intraperitoneal injection. However, this mode of administration lacks specificity for VSMC, and it remains uncertain whether K858’s inhibitory effects on neointimal formation after injury are attributed to reduced KIF11 activity in VSMC or the inhibition of systemic inflammatory responses. In future studies, we plan to investigate this by employing delivery methods that specifically target VSMC or by utilizing genetically modified mice with KIF11 knocked out in VSMC.

To sum up, this study provides the first evidence supporting KIF11 as a crucial mediator of VSMC cycle progression and proliferation. We have established the pivotal role of KIF11 in promoting VSMC cycle progression and proliferation. Notably, inhibiting KIF11 activity *in vivo* did not adversely affect the overall health of mice, suggesting that the side effects of therapeutic drugs targeting KIF11 may be limited if they exhibit sufficient specificity. Therefore, our findings offer potential therapeutic targets for neointimal formation.

## Data Availability

The data supporting the findings of this study are available within the article/supplementary material. The raw data has been uploaded to jianguoyun, available at: https://www.jianguoyun.com/p/DUY2RhAQp8W0DBjQwrgFIAA.
