# Supplementary material for: KIF11 promotes vascular smooth muscle cell proliferation by regulating cell cycle progression and accelerates neointimal formation after arterial injury in mice
Source: Front Pharmacol. 2024 Aug 6;15:1392352. doi: 10.3389/fphar.2024.1392352 (PMC11333341; doi:10.3389/fphar.2024.1392352)
Supplement: Supplementary file 1 [file Image1.PDF]

Supplementary Material

Supplementary Figures

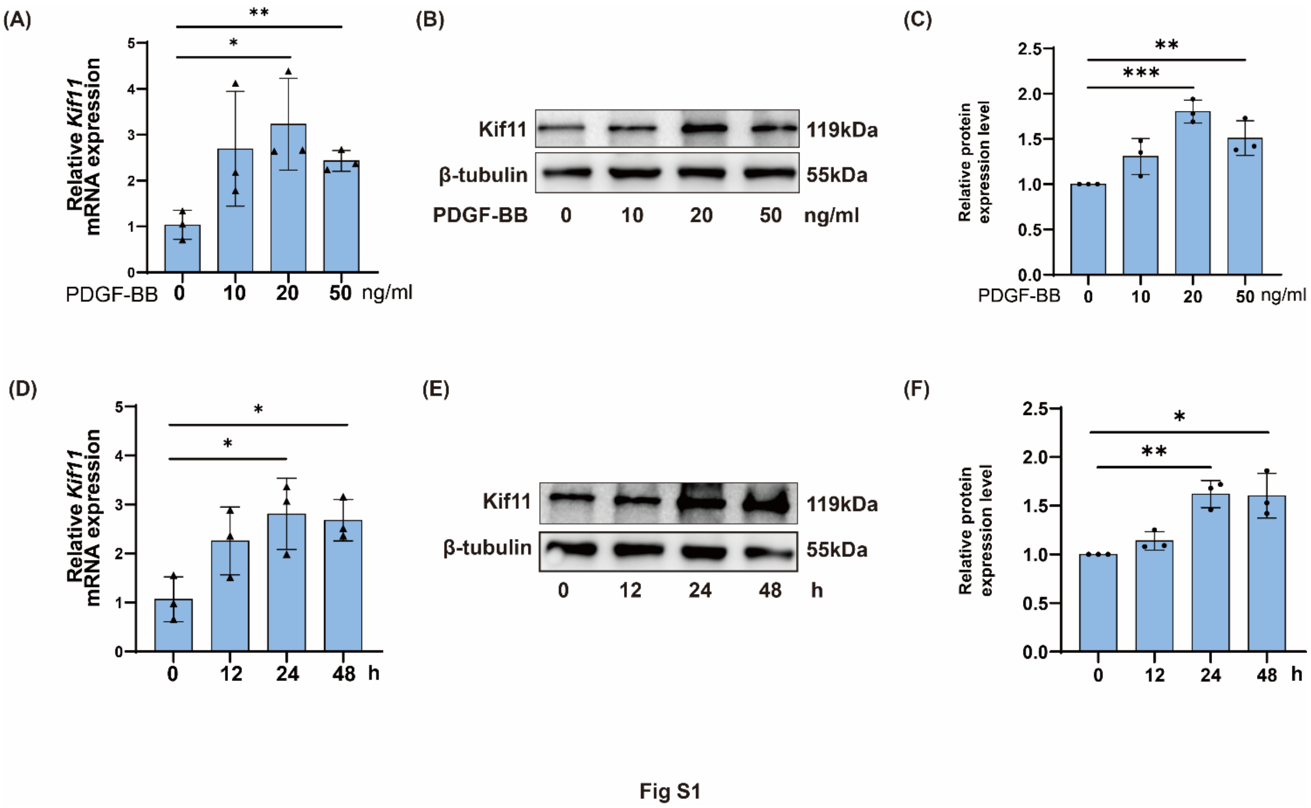

Supplementary Figure 1. (A-C) After treating VSMCs with different concentrations of PDGF-BB for 24 hours, use western blot and RT-PCR to assess the mRNA and protein expression levels of

KIF11. Bars (C) indicate the relative expression levels of the proteins. N=3. *p<0.05, **p<0.01, ***p<0.001.

(D-F) After treating VSMCs with PDGF-BB (20ng/ml), measure the mRNA and protein expression levels of KIF11 at different time points. Bars (F) indicate the relative expression levels of the proteins. N=3. *p<0.05, **p<0.01, ***p<0.001.

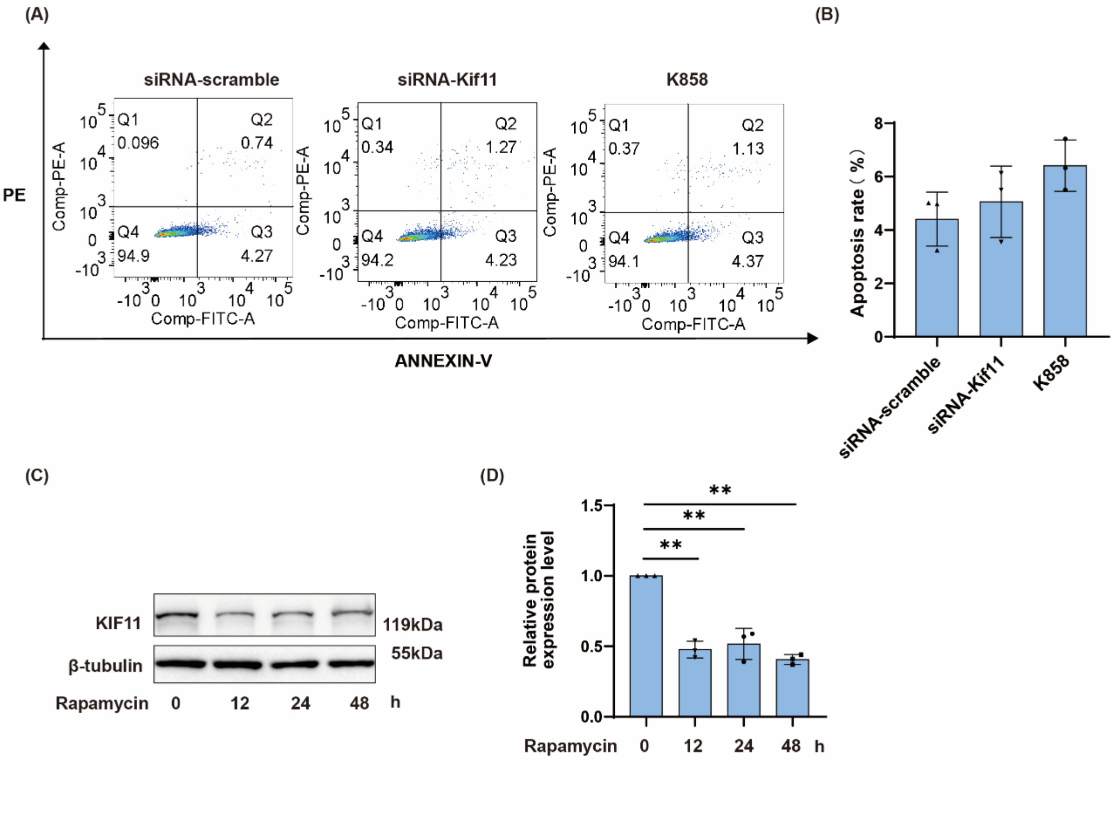

Supplementary Figure 2. (A-B) The apoptosis levels of the control group, siRNA-*Kif11* group and K858 group were counted by ANNEXIN-V flow cytometry. Bar chart (B) displays the proportion of cells in different cell cycle phases for the control and K858 groups. N=3. *p<0.05, **p<0.01, ***p<0.001.

(C-D) After treating VSMCs with different concentrations of rapamycin (20nM) for 24 hours, then treating the control group and rapamycin group VSMCs with PDGF-BB for 24 hours, use western blot assess the protein expression levels of KIF11. Bars (D) indicate the relative expression levels of the proteins. N=3. *p<0.05, **p<0.01.
